# *In situ* wet pharmaceutical granulation captured using synchrotron radiation based dynamic micro-CT

**DOI:** 10.1107/S1600577523000826

**Published:** 2023-02-17

**Authors:** Xiao Fan Ding, Sima Zeinali Danalou, Lifeng Zhang, Ning Zhu

**Affiliations:** a Division of Biomedical Engineering, University of Saskatchewan, 57 Campus Drive, Saskatoon, SK, S7N 5A9, Canada; b Department of Chemical and Biological Engineering, University of Saskatchewan, 57 Campus Drive, Saskatoon, SK, S7N 5A9, Canada; c Canadian Light Source Inc., 44 Innovation Blvd, Saskatoon, SK, S7N 2V3, Canada; RIKEN SPring-8 Center, Japan

**Keywords:** dynamic CT, *in situ*, micro-CT, time-resolved imaging, wet granulation

## Abstract

Synchrotron radiation based dynamic micro-computed tomography (micro-CT) is a powerful technique available at synchrotron light sources for investigating evolving microstructures. Wet granulation is the most widely used method of producing pharmaceutical granules, precursors to products like capsules and tablets. Granule microstructures are known to influence product performance, so this is an area for potential application of dynamic CT. Here, lactose monohydrate (LMH) was used as a representative powder to demonstrate dynamic CT capabilities. Wet granulation of LMH has been observed to occur on the order of several seconds, which is too fast for lab-based CT scanners to capture the changing internal structures. The superior X-ray photon flux from synchrotron light sources makes sub-second data acquisition possible and well suited for analysis of the wet-granulation process. Moreover, synchrotron radiation based imaging is non-destructive, does not require altering the sample in any way, and can enhance image contrast with phase-retrieval algorithms. Dynamic CT can bring insights to wet granulation, an area of research previously only studied via 2D and/or *ex situ* techniques. Through efficient data-processing strategies, dynamic CT can provide quantitative analysis of how the internal microstructure of an LMH granule evolves during the earliest moments of wet granulation. Here, the results revealed granule consolidation, the evolving porosity, and the influence of aggregates on granule porosity.

## Abstract 2

This article outlines the procedure for performing synchrotron radiation based dynamic micro-computed tomography of wet granulation of pharmaceutical powders and subsequent methods for quantitative analysis.

## 1. Introduction

Synchrotron radiation micro-computed tomography (micro-CT) has developed towards sub-second data acquisition (DAQ) speeds so that temporally evolving internal structures can be investigated non-destructively (García-Moreno *et al.*, 2021 ▸; Marone *et al.*, 2017 ▸; Mokso *et al.*, 2011 ▸; Villanova *et al.*, 2017 ▸). This capability, sometimes referred to as dynamic CT (Dewanckele *et al.*, 2020 ▸), is built upon developments over the past 20 years towards fast tomography, *in situ* imaging and time-resolved imaging (Bernard *et al.*, 2005 ▸; Chen-Wiegart *et al.*, 2012 ▸; Eggert *et al.*, 2014 ▸; Lame *et al.*, 2003 ▸). An area of research that could benefit from dynamic CT is wet granulation of pharmaceutical powders.

Wet granulation is the most widespread mode of granulation in capsule and tablet production. This process involves a liquid binder sprayed onto a pharmaceutical powder bed where countless droplets interact with the powders (Narang & Badawy, 2019 ▸; Shanmugam, 2015 ▸). A simplified model is used for wet-granulation research where a single liquid droplet is released onto the powders, which forms a single granule. This so-called ‘single-drop impact method’ is suitable for studying controlled granulation (Emady *et al.*, 2011 ▸; Hapgood *et al.*, 2003 ▸). The earliest stages of wet granulation are called wetting and nucleation (Iveson *et al.*, 2001 ▸). The granule’s internal structure that develops during wetting and nucleation can influence the final granule properties, which in turn can influence pharmaceutical product performance (Ban & Goodwin, 2017 ▸; Emady *et al.*, 2011 ▸; Li *et al.*, 2012 ▸; Poutiainen *et al.*, 2011 ▸).

Although internal structures are an area of interest, they are rarely investigated because the granule’s opaque appearance prevents direct observation (Li *et al.*, 2019 ▸). Previous wet-granulation studies have been more focused on external structures and formation mechanisms (Emady *et al.*, 2011 ▸, 2013 ▸; Gao *et al.*, 2018 ▸, 2020 ▸; Iveson *et al.*, 2001 ▸; Li *et al.*, 2019 ▸, 2021 ▸). There have been lab-based micro-CT studies on granule internal structures, but these have focused on the final granule product and not on *in situ* granulation (Crean *et al.*, 2010 ▸; Davis *et al.*, 2017 ▸; Farber *et al.*, 2003 ▸; Matsui *et al.*, 2019 ▸). The X-ray flux from lab-based scanners is not sufficient to capture *in situ* wet granulation (Li *et al.*, 2019 ▸). Although there have been *in situ* studies that used 2D projections to observe the powder and liquid interactions, these cannot provide any quantitative analysis of the 3D changes inside the granule (Li *et al.*, 2019 ▸, 2021 ▸).

Dynamic CT is a time-resolved 3D imaging technique that is non-destructive and does not require altering of the sample itself. Furthermore, as a synchrotron radiation based technique, image contrast can be enhanced with phase-retrieval algorithms (Paganin *et al.*, 2002 ▸). Dynamic CT can bring new insights to the *in situ* wet-granulation process in 3D, which has previously only been studied via 2D and/or 3D *ex situ* imaging techniques.

A basic principle among CT techniques is to acquire 2D X-ray projections of the sample around a central axis. To avoid motion artefacts, the sample’s structure is static during DAQ (Elliott & Dover, 1982 ▸; Iniewski, 2009 ▸; Romans, 2018 ▸). Dynamic CT keeps the conventions of acquiring projections around a central axis, but this time the sample’s structure is changing. To avoid motion artefacts during dynamic CT, there is a condition that the rate of change of the sample’s microstructure be sufficiently slow, *i.e.* within one detector pixel in the time for one scan (Maire *et al.*, 2016 ▸). To successfully scan a fast-deforming sample, even faster DAQ speed is required (Mokso *et al.*, 2010 ▸).

A research issue in dynamic CT is that the raw data are enormous, often upwards of 100 GB. For each raw dataset, it may be possible to perform CT reconstruction from tens of thousands of time points using a so-called ‘sliding-window’ reconstruction (García-Moreno *et al.*, 2019 ▸). Processing multiple datasets can quickly become a heavy task without a strategy at hand. Therefore, three data-processing strategies are introduced in this article for dealing with dynamic CT data, specifically for wet granulation. These strategies incorporate analysis methods used in studying pharmaceutical granules to enhance sliding-window reconstruction for efficient analysis of temporally evolving granules.

The objective of this article is to use dynamic CT to directly capture and quantify the changing internal structures during wet granulation. In addition, this article quantifies the loss in spatial resolution when reducing the number of projections acquired per CT (proj/CT), an often-cited compromise in dynamic CT literature (García-Moreno *et al.*, 2018 ▸; Marone *et al.*, 2020 ▸). The experimental and data-processing procedures presented allowed for novel 3D *in situ* investigation of the changing internal structure, chiefly the porosity, during the earliest moments of wet granulation. The success of this article serves as a framework and guide towards future investigations with dynamic CT in pharmaceutical and chemical engineering research.

## 2. Materials and methods

### 2.1. Wet-granulation experiment

**Figure 1:**
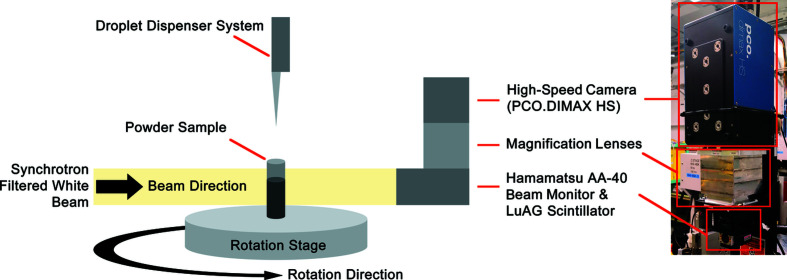
The experimental setup for performing *in situ* wet granulation. The sample on top of the rotation stage was positioned between the X-ray source and detector and below the droplet dispenser system.

The wet granulation of lactose monohydrate (LMH) as a representative pharmaceutical material was scanned using dynamic CT. The wet-granulation experiment was performed *in situ* during the scan by releasing a 15 µl droplet of deionized water positioned 2.5 cm above the powder bed. The droplet was released by a dispenser system which consisted of a micropipette connected to a timed syringe pump. The timed system allowed sufficient time for the researchers to safely exit the experimental hutch, start the sample rotation stage, and take control of the imaging system before the droplet would be released from the pipette. A schematic diagram of inside the experimental hutch is shown in Fig. 1 ▸. An additional scan raised the drop height to 15 cm because an increased drop height has been observed to change the mechanism by which the granule forms (Emady *et al.*, 2011 ▸; Li *et al.*, 2019 ▸).

The pharmaceutical samples were loaded into cylinder containers (Fisher Scientific International Inc., USA) and mounted onto an air-bearing rotation stage (Aerotech Inc., USA). Each dynamic CT scan involved one cylinder at a time. The distance from the X-ray source to the sample was 26 m and the distance from the sample to the detector was 50 cm. The sample-to-detector distance is one of the parameters for phase-retrieval algorithms to enhance image contrast (Paganin *et al.*, 2002 ▸; Wilkins *et al.*, 1996 ▸).

DAQ involved several manual steps. The undisturbed powder bed was first put into free rotation at a constant speed. The high-speed camera was turned on and began collecting projections in free-running mode. The camera’s internal memory allowed for 20 s of recording before the earliest projections were overwritten. Once the droplet dispenser system was turned on, it was closely monitored. To ensure that the earliest data would not be overwritten, the camera was stopped at ∼18 s after the droplet was released. This mode of data collection has been called ‘buffer mode’ (Maire *et al.*, 2016 ▸).

### 2.2. Synchrotron dynamic CT setup

Dynamic CT scans of *in situ* wet granulation were performed at the 05B1-1 beamline at the Canadian Light Source. The X-ray source was a white beam filtered through 0.8 mm of aluminium filter. The peak energy was around 20 keV. Indirect detection was used consisting of an AA-40 beam monitor (Hamamatsu Photonics K.K., Japan) attached to a 200 µm-thick LuAG scintillator. Images were captured by a PCO.DIMAX HS4 camera (PCO AG, Germany). The effective pixel size of the detector was 5.5 µm.

Each dynamic CT raw dataset consisted of a continuous sequence of 20000 projections that were collected on the fly. The DAQ speed was 500 projections per 180° rotation of the sample, with no delay between projections. The field of view (FOV) for each projection was 11 mm × 4.4 mm. The exposure time was 1 ms. The exposure time was an optimized choice found by trial and testing. If a shorter exposure was used, the reduced signal would result in poorer signal-to-noise ratio; whereas, if a longer exposure time was used, the temporal resolution would be slower and therefore compromise capturing *in situ* wet granulation.

The frame rate of the high-speed camera was calculated by taking the reciprocal of the exposure time because each X-ray projection acquired is synonymous with a frame captured by the camera. The frame rate of this study comes to 1000 frames s^−1^. The rotation speed of the sample was calculated from the frame rate and the number of proj/CT, as demonstrated in equation (1). The rotation speed of this study was 360° s^−1^,

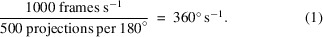




### 2.3. Spatial resolution test

The number of projections, *N*_projections_, for a π rotation of the sample can be calculated from the distance extending from the centre of rotation, radius (*R*) and the pixel size (*p*) (Joseph & Schulz, 1980 ▸; Kak & Slaney, 2001 ▸),

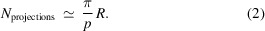

For the FOV width of 11 mm used in this study, *N*_projections_ ≃ 3000 by equation (2). However, only 500 projections were acquired during actual *in situ* experiments. The choice to reduce the number of proj/CT rotation for faster DAQ speed is an often-cited compromise (García-Moreno *et al.*, 2018 ▸; Marone *et al.*, 2020 ▸). A bar pattern phantom (QRM GmbH, Germany) was scanned at 3000, 500 and 100 proj/CT to demonstrate the deterioration in spatial resolution with fewer projections. The spatial resolution for each scan of the phantom was calculated from the modulation transfer function (MTF) at discrete spatial frequencies (Sharma *et al.*, 2010 ▸). The resolution at the 10% MTF is considered the limit of human vision (Ghani *et al.*, 2016 ▸; Langner *et al.*, 2009 ▸; Sun *et al.*, 2022 ▸).

### 2.4. Dynamic CT data-processing strategies

Dynamic CT data were acquired while wet granulation was performed *in situ*. CT reconstruction could be performed with any 500 consecutive projections because the sample was continuously rotating during DAQ. The intrinsic temporal resolution of each CT was 500 ms. The time interval between any two CTs is equivalent to the time elapsed between each CT’s first projection. This mode of CT reconstruction has been called sliding-window reconstruction (Rasche *et al.*, 1995 ▸; Zanette *et al.*, 2012 ▸).

**Figure 2:**
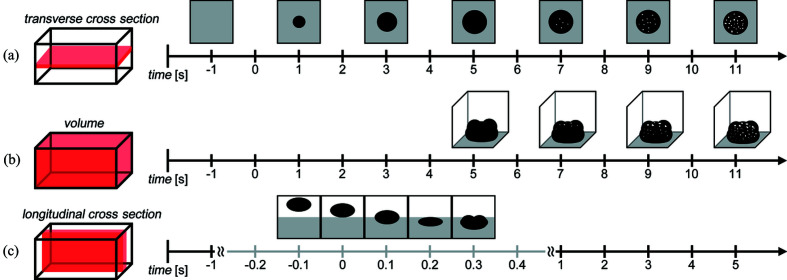
A schematic drawing of three data-processing strategies for organizing the raw data before CT reconstruction. As an illustration, the time points are not indicative of any scan. (*a*) Transverse cross-sectional views to capture the temporally evolving granule at different regions of the granule (strategy A). (*b*) The whole volume and 3D analysis of evolving porosity inside the granule (strategy B). (*c*) Longitudinal cross-sectional views extracted from whole volumes to capture processes with vertical movement (strategy C).

The three data-processing strategies that were used are shown in Fig. 2 ▸ for dynamic CT of wet granulation. Strategy A outlines examining transverse cross-sectional views, which allows for fast processing time at small time intervals for local variations in porosity at different vertical positions of the granule. Strategy B follows 3D analysis of the granule’s overall porous structure. Strategy C examines longitudinal cross-sectional views to capture the vertical movement of liquid onto/through the powder bed with quantitative analysis in 3D.

Time-zero was defined as the time when the droplet impacted on the powder bed and the wetting part of wet granulation began. Time-zero varied from scan to scan and needed to be manually determined from each raw dataset because DAQ was manually started and stopped.

### 2.5. Software used

Original Python scripts were written to organize raw data according to the strategies of Fig. 2 ▸. CT reconstruction was performed with *UFO-KIT* based software (Faragó *et al.*, 2022 ▸; Paganin *et al.*, 2002 ▸; Vogelgesang *et al.*, 2016 ▸). *ImageJ* (Rasband, 2012 ▸) was used for post-processing, such as reorienting the region of interest (ROI), thresholding, and binarizing images to measure physical features of the granules. *Dragonfly* (Objects Research Systems Inc., Canada) was used for 3D rendering (ORS, 2021 ▸). *MATLAB* (The MathWorks Inc., USA) was used to create graphical results.

## 3. Results and discussion

### 3.1. Spatial resolution in dynamic CT

**Figure 3:**
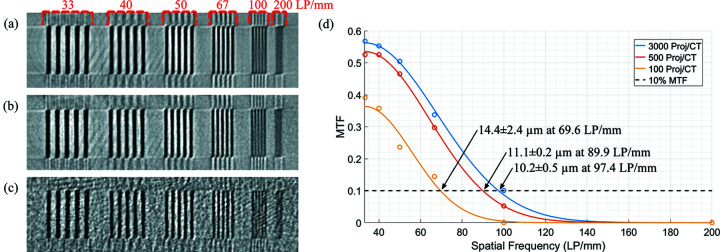
CTs of bar patterns with 3000, 500 and 100 proj/CT are shown in (*a*), (*b*) and (*c*), respectively. The bar patterns increase in spatial frequency from 33 to 200 Lp mm^−1^. The fit of the MTF from discrete spatial frequencies and the spatial resolution at the 10% MTF are shown in (*d*).

It is known that the resultant granules of similar materials have pores that are 10 to 100 µm in diameter (Farber *et al.*, 2003 ▸). Therefore, a spatial resolution of 10 µm was the minimum visible requirement of the imaging system. Visually, the spatial resolution at 3000 proj/CT, Fig. 3 ▸(*a*), was less noisy than the spatial resolution at 500 proj/CT, Fig. 3 ▸(*b*). In both, the 100 line-pairs per millimetre (Lp mm^−1^) patterns were partially resolved and the 67 Lp mm^−1^ patterns were fully resolved. At 100 proj/CT, shown in Fig. 3 ▸(*c*), the 100 Lp mm^−1^ patterns became indistinguishable from noise. Quantitatively from Fig. 3 ▸(*d*), the spatial resolutions of the imaging system at 3000, 500 and 100 proj/CT with 95% confidence were 10.2 ± 0.5, 11.1 ± 0.2 and 14.4 ± 2.4 µm, respectively. This shows that using 500 proj/CT can visualize the majority of pores.

There were two reasons for reducing the number of projections from 3000 to 500. The first was that the focus of this study was the porous structure within the granule. Since it was known that most pores are larger than 10 µm, using 500 proj/CT could reliably produce satisfactory results. The shape of the granule could be identified from the characteristic gap that forms by droplet impact, which spans 30–40 µm (Li *et al.*, 2019 ▸). The second reason was that the number of projections directly affects the rotation speed, *i.e.* a 3000 proj/CT scan must physically rotate slower than 500 proj/CT for the same exposure time.

**Figure 4:**
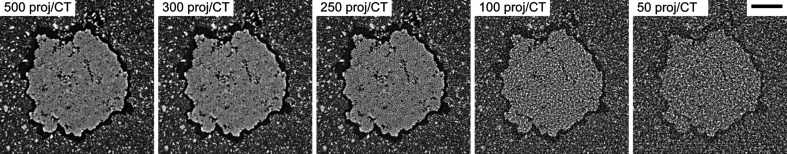
A demonstration of deterioration in spatial resolution with fewer and fewer proj/CT on an image of a granule. The scale bar represents 1 mm.

The deterioration in spatial resolution with fewer projections was demonstrated on granule data, as shown in Fig. 4 ▸. There is potential to use a DAQ speed of 300 or 250 proj/CT because the spatial resolution is still sufficient to distinguish and measure the pores within the granule. At 100 proj/CT is the limit where noise prevents accurate measurement of the pores, but the granule shape could still clearly be seen by the gap. At 50 proj/CT, the image has become too noisy to accurately measure the granule shape.

For dynamic CT to expand into more diverse granulation experiments, the capability and potential of using fewer projections and faster DAQ speeds need to be understood. There are several factors which may influence the rate of granulation, and situations that require faster DAQ speeds are within the realm of possibilities. The results in Fig. 4 ▸ are a step towards mature dynamic CT applications to wet granulation.

### 3.2. Evolving granule cross sections

**Figure 5:**
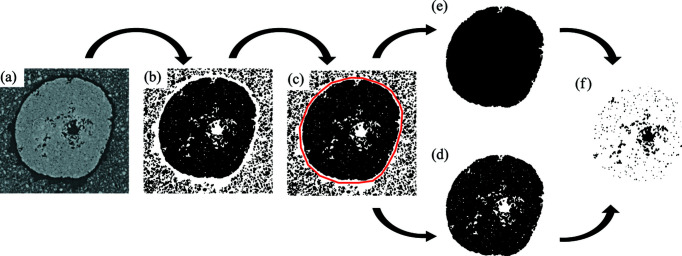
A flowchart beginning with the CT image to segment out the granule and pores. (*a*, *b*) Starting with the CT image, a threshold was applied to create a binarized mask. (*c*) A polygon was drawn using the gap between the granule and powder bed to segment the granule. (*d*, *e*) From the segmented granule, two masks, one including the pores and the other without, were made. (*f*) A mask of the pores alone was generated with the exclusive pixel information.

Strategy A followed temporally evolving transverse cross-sectional views of the granule with a time interval of 100 ms. The gap formed between the granule and the powder bed upon impact of the water droplet was used to segment the granule in each cross section, as shown in Fig. 5 ▸. Over the 20 s observed, the gap between the granule and the powder bed expanded as the granule contracted, so the polygon drawn in Fig. 5 ▸(*c*) to segment the granule could be reliably reused at other time points.

**Figure 6:**
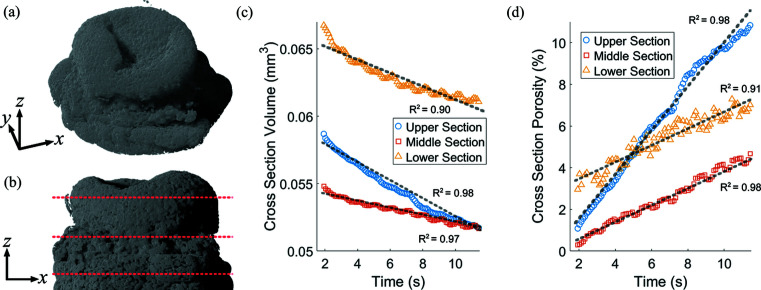
Three equidistant cross-section volumes in the upper, middle and lower sections of an LMH granule were chosen and are shown in a 3D model, with the orthogonal view shown in (*a*) and the front view shown in (*b*). The cross-sectional volumes and porosities are plotted in (*c*) and (*d*), respectively. Supporting videos of each cross section are provided and are titled ‘sc2-upper’, ‘sc2-middle’, and ‘sc2-lower’.

Cross-sectional volumes at different vertical positions of a granule and associated porosities over time are plotted in Figs. 6 ▸(*c*) and 6 ▸(*d*). The cross sections were in the upper, middle and lower regions of the granule, which serve as representative conditions and overall changes of their respective regions (Gao *et al.*, 2018 ▸). The volumes and porosities were calculated by first binarizing the images and summing the pixels, which constituted the granule area and the void area within the granule (pores). The porosity is the percentage of void volume to total volume.

The cross-sectional volume and porosity trended in opposite directions, which reflected liquid binder dissipating throughout the granule. The greater volume at a lower position of the granule is because LMH granules become imbedded in the powder bed after wet granulation. Furthermore, because the upper layer was directly exposed to air, evaporation could have affected the faster rate of change in porosity. This suggests that there is local variation in the rate at which pores form, *i.e.* closer to the surface or closer to the powder bed. This variation could be explored by applying dynamic CT and strategy A to more diverse pharmaceutical materials and experimental conditions.

The sliding-window reconstruction produces a smoothing effect at time intervals under the intrinsic temporal resolution due to overlapping data points (Rasche *et al.*, 1995 ▸; Zanette *et al.*, 2011 ▸, 2012 ▸). For this study, the evolution of pores as shown in Figs. 6 ▸(*c*) and 6 ▸(*d*) was linear enough, *R* ^2^ ranging from 0.90 to 0.98 (where *R* ^2^ relates to goodness of fit), that such smoothing did not influence observations. The trajectory of evolving porosity did not differ from when analysed using time intervals where the smoothing was absent.

### 3.3. Evolving porosity in the granule

**Figure 7:**
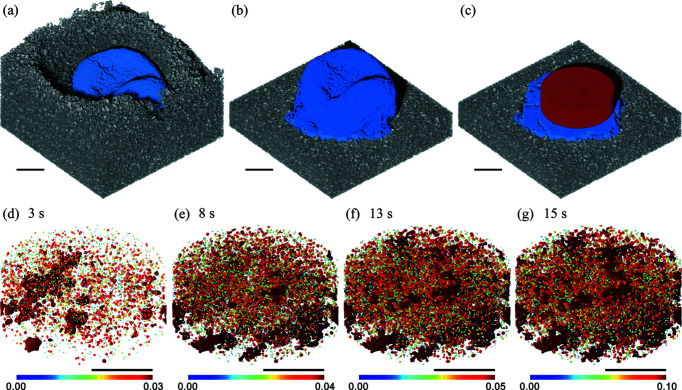
Three-dimensional renders of an LMH granule after 15 s into the wet-granulation process are coloured in blue and the surrounding powder bed is coloured in grey in (*a*) and (*b*). A cylinder representation of the granule’s internal structure is coloured in red and is shown in (*c*). Three-dimensional renders of the internal pores at four time points are shown in (*d*)–(*g*). The colour bar represents the volume of the pores in mm^3^. The black scale bar is 1 mm in every image.

Strategy B was used for analysing the evolution of porosity and pores after the granule has consolidated. Segmenting the granule from the surrounding powders followed the procedure shown in Fig. 5 ▸ but performed for every slice. A volume within the granule was extracted and analysed to be representative of the internal structures of the granule (Gao *et al.*, 2018 ▸). 3D renderings of the granule, surrounding powders and representative cylinder are shown in Figs. 7 ▸(*a*)–7 ▸(*c*). The pores within the representative volume at four time points are shown in Figs. 7 ▸(*d*)–7 ▸(*g*).

**Figure 8:**
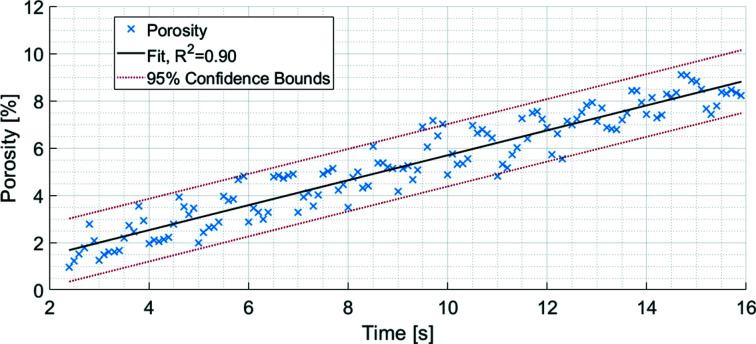
The evolving porosity of an LMH granule with 95% confidence bounds. The fit of the porosity data shows a linear trend that has an *R* ^2^ of 0.90. Analysed using a 100 ms time interval, the data points are separated by 100 ms.

A plot of the evolving granule porosity at a 100 ms time interval is shown in Fig. 8 ▸. Like that carried out in Fig. 7 ▸, a representative volume was extracted from the granule to calculate the porosity. With 95% confidence bounds, the linear fit of evolution of porosity has an *R* ^2^ of 0.90. The goodness of fit shows confidence that the evolution in porosity in the LMH granule during the first 15 s of wet granulation is linear.

The changing porosity, pore count, and total and mean volumes are listed in Table 1 ▸. The mean pore volume was of the order of 10^−5^ mm^3^, whereas the total pore volume was of the order of 10^−1^ mm^3^. The number of pores may decrease with time, while the total pore volume increased. This suggests that smaller pores over time have consolidated into larger pores. The porosity progressed from 2 to 9% over the observed time points.

The results from Fig. 8 ▸ and Table 1 ▸ may not have captured the evolution of pores until the end. Since this study has revealed that the evolution of pores is possibly a linear process, time-lapse imaging could be considered as a follow up. Time-lapse imaging would not allow analysis using time intervals but it would allow for scanning longer processes.

Furthermore, each dynamic CT scan was followed by ∼25 minutes of readout time during which another scan cannot be taken. Given the observed linearity in the evolution of pores thus far, imaging in step-and-shoot mode or writing raw data directly to disk could be considered (Kak & Slaney, 2001 ▸). These imaging options would eliminate the long readout time and be suitable for granulation experiments with even slower dynamics than the LMH granulation shown in this study.

Conversely, granulation studies with faster dynamics would be harder to deal with. The evolution of pores in the LMH was relatively slow compared with the DAQ speed, which satisfied the condition that the dynamics be within one detector pixel per scan. Should the evolution of pores be faster, there would be more intrusive motion artefacts. In such cases, approximation models are required to relax the one detector pixel condition (Nikitin *et al.*, 2019 ▸; Ruhlandt *et al.*, 2017 ▸).

### 3.4. Droplet–powder interactions

Strategy C captured wet granulation in longitudinal cross-sectional views and whole volumes for quantitative analysis at a 100 ms time interval. The longitudinal cross sections showed vertical movement that would not be obvious in transverse cross sections. There were two experiments where this was the case. In one, the droplet descent into the powder bed was clearly visible and measured. In the other, the initial powder bed was inhomogeneous and contained large aggregates. The liquid dissipating through the aggregate was captured and the variations in granule porosity quantified.

**Figure 9:**
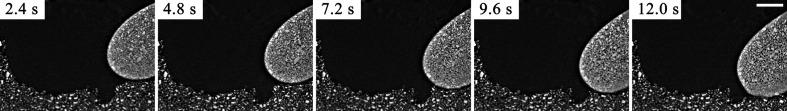
Longitudinal cross sections capturing the droplet descending slowly. The scale bar represents 1 mm. A supporting video of this process titled ‘sc11-XZ’ is provided.

The progression of a droplet descending when the drop height was raised to 15 cm is shown in Fig. 9 ▸. It was observed that the rate of granulation became significantly slower when releasing a droplet from 15 cm. The average speed of a droplet descending into the powder bed was 1.27 ± 0.28 mm s^−1^ when the drop height was 2.5 cm. At a 15 cm drop height, the droplet descended at a speed of 0.074 mm s^−1^. This slower rate may be a consequence of different granulation mechanisms, which occur at different drop heights.

**Figure 10:**
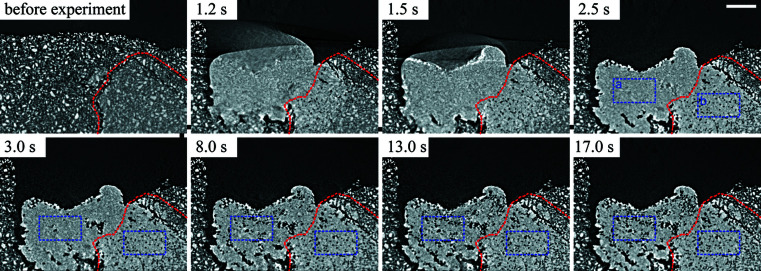
Longitudinal cross sections at various time points capturing droplet interaction with an aggregate, outlined in red, and the resulting granule with regions of differing porosity. Two ROIs are outlined in blue and labelled ‘a’ and ‘b’. The scale bar represents 1 mm. A supporting video of this process titled ‘sc4-YZ’ is provided.

**Figure 11:**
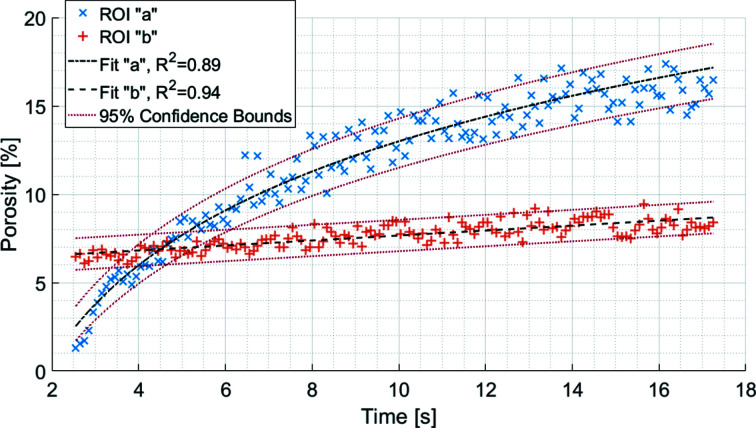
The evolving porosity of ROIs ‘a’ and ‘b’ with 95% confidence bounds. Fits ‘a’ and ‘b’ are the fitted curves to ROIs ‘a’ and ‘b’, respectively. The curves show how, in the presence of an aggregate, the resulting granule can have variations in porosity. The data points are separated by 100 ms.

An LMH granule forming in an inhomogeneous powder bed is shown in Fig. 10 ▸. The liquid travelling through an aggregate is visible from the change in grey value in the first few seconds. After the granule consolidated at around 2.5 s, two regions of differing porosity emerged. Two ROIs labelled ‘a’ and ‘b’ were extracted as representative volumes for regions outside and inside the aggregate, respectively. The evolving porosities of the two ROIs are shown in Fig. 11 ▸.

The regions inside and outside of the aggregate not only possessed different porosities but also possessed different rates of increasing porosity. The porosity inside the aggregate, ROI ‘b’, was consistent over time as the microstructure was well established earlier on in the time sequence. The porosity outside of the aggregate, ROI ‘a’, showed a steep increase and then levelling off. This resembles a logarithmic growth curve and was fitted as such. The presence of an aggregate increased the rate at which the liquid binder dissipated in the first 16 s of wet granulation before levelling off as the main driving force behind porosity changed from granulation to evaporation. Inhomogeneous powder beds are a separate area of interest for pharmaceutical research (Liu *et al.*, 2013 ▸; Oka *et al.*, 2015 ▸). The results of Fig. 11 ▸ show potential for dynamic CT to explore the influence of inhomogeneous powder beds and the presence of aggregates.

The porosity curves shown in Fig. 11 ▸, and similarly in Figs. 8 ▸ and 6 ▸, are not straight curves depicting the evolving porosity. While it would be ideal to be able to simply track how granule porosity changes over time in an unbroken line, it remains a challenge. The oscillations and variability could be the result of several factors, *i.e.* imaging, physical or chemical. Wet granulation is a system of loose particles disrupted by a liquid droplet. To perform dynamic CT on wet granulation *in situ* requires accelerating an already complex system to a fast rotation speed. It may even be possible that the granule is experiencing some motion on the submicrometre scale, considering there are no obvious motion artefacts in the data. There is visible space between the granule and the surrounding powder bed, as shown in the supporting videos. At present, dynamic CT with the imaging parameters in this article has shed light on how porosity evolves during the earliest moments of wet granulation, but it has not revealed the full story. With further enthusiasm in studying the relationship between granule microstructure and dissolution (Ansari & Stepanek, 2008 ▸), powder mixing (Oka *et al.*, 2015 ▸), and granule drying (Li *et al.*, 2022 ▸), each phenomenon may present their own post-processing challenges when investigated with dynamic CT.

## 4. Conclusions

Dynamic CT was used to capture and quantify the evolving porosity of LMH granules. This was carried out non-destructively and without alterations to the pharmaceutical material. The enormous raw dataset from dynamic CT can be difficult to process so data-processing strategies were useful when processing dynamic CT of wet granulation. Strategy A analysed temporally evolving transverse cross sections at different regions of the granule. Strategy B followed 3D renders of the granule for quantitative analysis of granule porosity over time. Strategy C captured the droplet–powder interactions and the influence of large aggregates on granule porosity. Since the porous structure of granules is known to influence pharmaceutical product performance, the success of quantifying wet granulation of a common material like LMH is a critical first step towards investigation of more diverse pharmaceutical formulations and experimental conditions. The analytical approaches with dynamic CT presented in this article can serve as a framework to bring new insights to pharmaceutical granulation research.

## Supplementary Material

Supporting video sc2-upper. DOI: 10.1107/S1600577523000826/yi5128sup1.mp4

Supporting video sc2-middle. DOI: 10.1107/S1600577523000826/yi5128sup2.mp4

Supporting video sc2-lower. DOI: 10.1107/S1600577523000826/yi5128sup3.mp4

Supporting video sc11-XZ. DOI: 10.1107/S1600577523000826/yi5128sup4.mp4

Supporting video sc4-YZ. DOI: 10.1107/S1600577523000826/yi5128sup5.mp4

## Figures and Tables

**Table 1:** Pores and porosities of LMH wet granulation at corresponding time points from Fig. 7 ▸

Time (s)	Porosity (%)	Total pore count	Total pore volume (mm^3^)	Mean pore volume (mm^3^)
3	2.08	4305	0.14	2.79 × 10^−5^
8	6.45	8311	0.42	4.09 × 10^−5^
13	7.93	9171	0.52	5.23 × 10^−5^
15	8.88	8349	0.58	6.83 × 10^−5^
